# A Method of Three-Dimensional Recording of Mandibular Movement Based on Two-Dimensional Image Feature Extraction

**DOI:** 10.1371/journal.pone.0137507

**Published:** 2015-09-16

**Authors:** Fusong Yuan, Huaxin Sui, Zhongke Li, Huifang Yang, Peijun Lü, Yong Wang, Yuchun Sun

**Affiliations:** 1 Center of Digital Dentistry, Peking University School and Hospital of Stomatology, Beijing, China; 2 Faculty of Prosthodontics, Peking University School and Hospital of Stomatology, Beijing, China; 3 National Engineering Laboratory for Digital and Material Technology of Stomatology, Beijing, China; 4 Research Center of Engineering and Technology for Digital Dentistry, Ministry of Health, Beijing, China; 5 The Second Artillery Engineering College, Xi’an, China; University of Chinese Academy of Sciences, CHINA

## Abstract

**Background and Objective:**

To develop a real-time recording system based on computer binocular vision and two-dimensional image feature extraction to accurately record mandibular movement in three dimensions.

**Methods:**

A computer-based binocular vision device with two digital cameras was used in conjunction with a fixed head retention bracket to track occlusal movement. Software was developed for extracting target spatial coordinates in real time based on two-dimensional image feature recognition. A plaster model of a subject’s upper and lower dentition were made using conventional methods. A mandibular occlusal splint was made on the plaster model, and then the occlusal surface was removed. Temporal denture base resin was used to make a 3-cm handle extending outside the mouth connecting the anterior labial surface of the occlusal splint with a detection target with intersecting lines designed for spatial coordinate extraction. The subject's head was firmly fixed in place, and the occlusal splint was fully seated on the mandibular dentition. The subject was then asked to make various mouth movements while the mandibular movement target locus point set was recorded. Comparisons between the coordinate values and the actual values of the 30 intersections on the detection target were then analyzed using paired t-tests.

**Results:**

The three-dimensional trajectory curve shapes of the mandibular movements were consistent with the respective subject movements. Mean XYZ coordinate values and paired t-test results were as follows: X axis: -0.0037 ± 0.02953, P = 0.502; Y axis: 0.0037 ± 0.05242, P = 0.704; and Z axis: 0.0007 ± 0.06040, P = 0.952. The t-test result showed that the coordinate values of the 30 cross points were considered statistically no significant. (P<0.05)

**Conclusions:**

Use of a real-time recording system of three-dimensional mandibular movement based on computer binocular vision and two-dimensional image feature recognition technology produced a recording accuracy of approximately ± 0.1 mm, and is therefore suitable for clinical application. Certainly, further research is necessary to confirm the clinical applications of the method.

## Introduction

Mandibular movement can be measured using track points, curves and condylar and incisal path inclination. Data from these and other key parameters are used in the diagnosis of oral disorders and in the research and design of the functional occlusal surfaces of dental prostheses. And knowledge of the mandible movement is helpful for a better understanding of the normal function of the temporomandibular joint (TMJ), and for the study of the aetiology, diagnosis and subsequent treatment of temporomandibular disorders.[1–2] It also has a profound influence on the development of articulators and on the evaluation of the health of the masticatory system.[3–5] Therefore, accurate measurement of the three-dimensional (3D) motion of the mandible relative to the maxilla is essential for relevant clinical applications. Physiological and pathological occlusion can be analyzed by first recording mandibular movement in three dimensions and then simulating this movement digitally. This technique can also be utilized to personalize the design of the occlusal surface of a dental prosthesis, which could improve the efficiency of clinical diagnosis and treatment and the quality of the dental prosthesis for the patient. [6]

3D mandibular movements were first recorded using a complicated and bulky device with two mechanical face bows.[7–8] Since then, much more compact and delicate systems have been developed, making significant contributions to clinical dentistry. However, most commercially available mandibular movement recording apparatuses install their signal transmitting or receiving devices directly on the soft tissue of the head or face, which results in recording errors due to the instability of the soft tissue [9–11]. Transoral measurement devices attached directly to the teeth have been used to remove skin movement artefacts, but these devices might interfere with the jaw movements. Therefore, there is a need for an accurate method for measuring 3D mandibular motion for clinical and research purposes without using devices that may impede jaw movements. Using specialized two-dimensional images and binocular stereo vision measurement technology, this study seeks to establish and evaluate a high-speed, high-precision hardware and software platform to measure mandibular movement. This system could therefore be used to also improve the accuracy of occlusal surface design for dental prostheses. With these important characteristics, the system could be suitable to assist dentists in their diagnosis and treatment plans, in particular in relation to oral rehabilitation treatments; the system may allow the diagnosis of clinical pathologies in the temporomandibular joints and precise occlusal surface design.

## Materials and Methods

### Materials

Hardware: Industrial digital camera, MV-130UM, gray monochrome 1024, resolution: 1280 × 1024 ppi, USB2.0 interface (Microvision, Xi’an, China); 16-mm focal length lens, resolution: 1,000,000 pixels(Seiko, Tokyo, Japan); E-SUTDIO506 A3 black and white digital copiers, DP-5010, print resolution: 2,400 × 600 dpi(Toshiba, Tokyo, Japan).

Software: Camera supporting SDK development package developed using Visual Basic 6.0(Microsoft, Redmond, USA); Geomagic Studio 2012 (Geomagic, Morrisville, USA); Imageware 13.0 (Electronic Data Systems, Plano, USA)

Other: A pair of complete natural dentition models; hard dental diaphragm, 1-mm thick (Erkodent, Pfalzgrafenweiler, Germany); light-cured temporal denture base resin material, Megatray Base Plate (Megadenta, Radeburg, Germany); printing paper, 0.1-mm thick.

### Methods

#### Ethics statement

This study was approved by the Bioethics Committee of the Stomatological Hospital of Peking University, Beijing, China. (No.PKUSSIRB-201412008. Date: 18/6/2014). Written informed consent to publish these case details was obtained from all subjects, and all procedures were approved by the Bioethics Committee. The individual in this manuscript has given written informed consent (as outlined in PLOS consent form) to publish these case details.

#### Building of a hardware platform for a mandibular three-dimensional trajectory recording system

Two main components were included to form the hardware system: the core computer binocular vision device with two digital cameras and a head-retention bracket affixed to that device. To track mandibular movement trajectory, the subject’s head was fixed on the head-retention bracket, and a detection target was rigidly fixed on the subject’s lower dentition outside of the mouth. As soon as the subject’s mandible began to move, the cameras began capturing images of the target at a speed of 10 frames per second. These data were then transmitted into the software to calculate the target’s position and orientation information in real time.

The trajectory acquisition software was developed using Visual Basic 6.0. Two graphics windows were open on the computer screen: a smaller one on the left, which displayed a cube graphic, and a larger one on the right, which displayed real-time images taken by the camera and overlaid identifications of the target and tracking signs of the graphic. When the target made a translation or a rotation movement, the cube graphic would mimic this movement, presenting an intuitively-expressed method of sample tracking.

**Fig 1:**
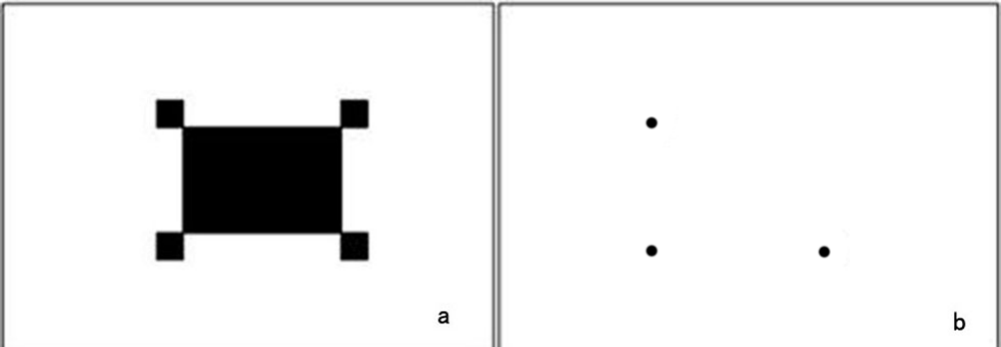
Design of the target. (a) a piece of white printing paper having one 32 mm × 23.5-mm black rectangle and four 4 mm × 4-mm black squares; (b) The three dots represent the three targets (the lower left corner, the lower right corner and the upper left corner) in the overall detection target.

The detection target was a piece of white printing paper having one 32 mm × 23.5-mm black rectangle and four 4 mm × 4-mm black squares that was pasted to a flat plastic plate. The goal of the working hardware and software was to rapidly extract the pixel coordinates of three corners of the detection target area (Fig 1).

#### Recording of mandibular trajectory points and subsequent three-dimensional reconstruction

**Fig 2:**
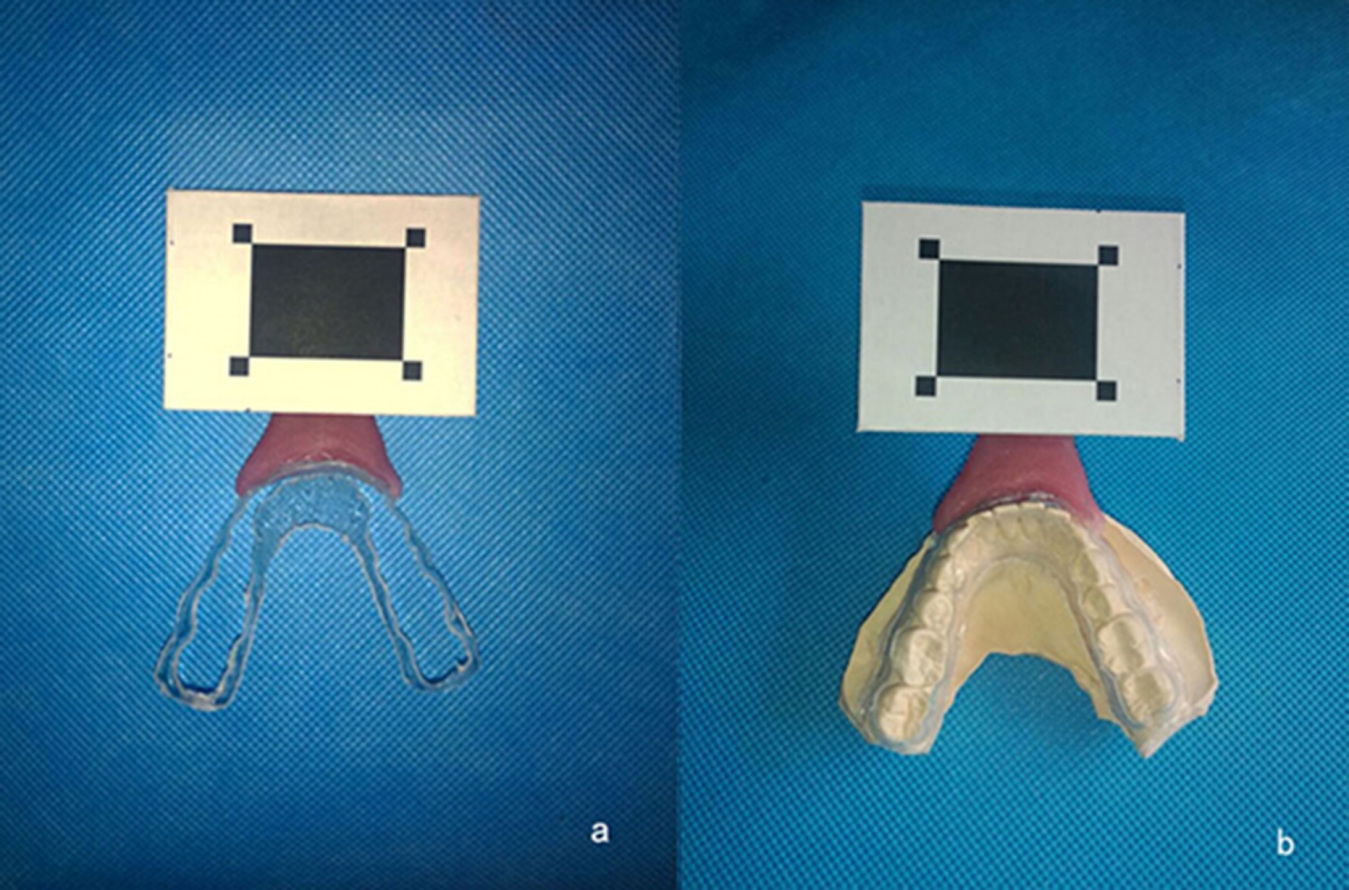
Mandibular occlusal splint. (a) Hard lower occlusal splint without occlusal surface; (b) Mandibular occlusal splint fully seated on the plaster model.

The upper and lower dentition plaster model was made using conventional methods. A mandibular occlusal splint was then made on the plaster model, and the portion of occlusal surface was removed. (Fig 2)

**Fig 3:**
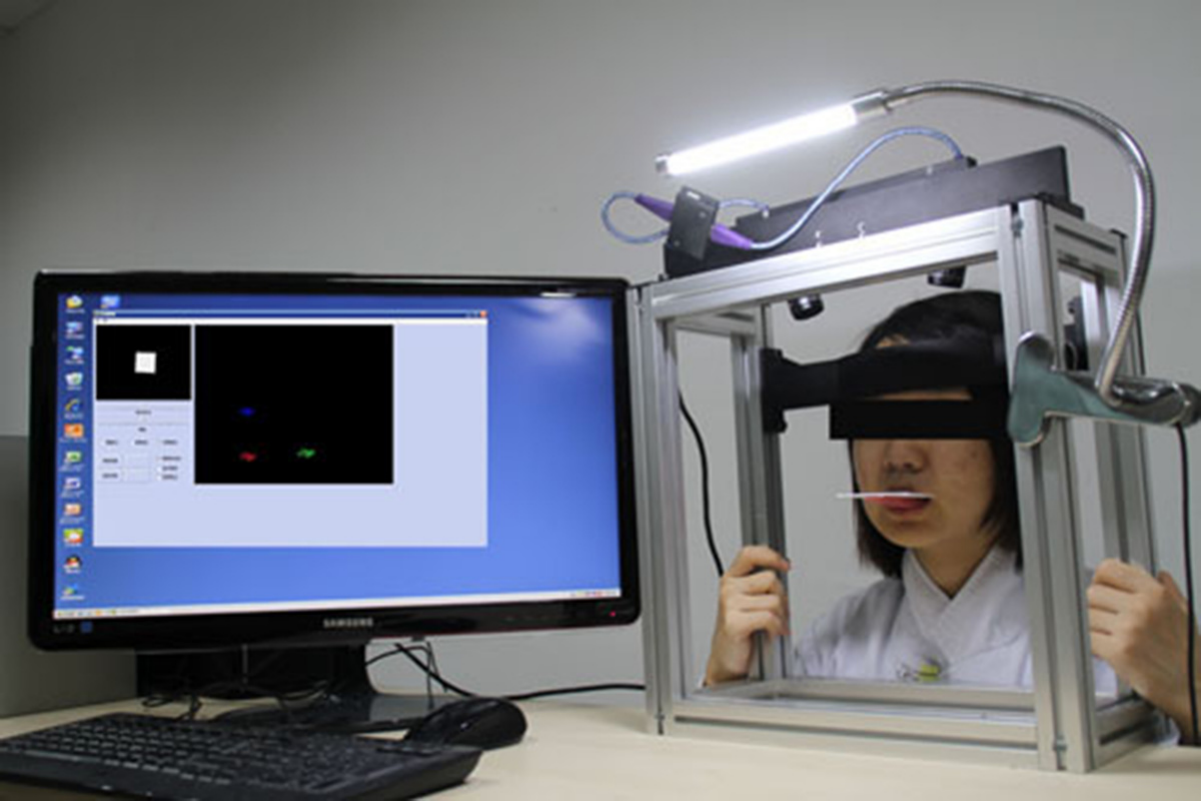
The process of recording mandibular movement.

Firstly, a square, flat piece of plastic was made and attached to both the labial surface of the anterior splint and a resin handle connecting the plastic and the splint. The detection target was then bonded to the surface of the handle. The subject was measured on the experimental platform, then a pair of clinical plaster models of the complete dentition were made, producing a mandibular splint without occlusal surface. The anterior region of the labial surface of the splint was bonded to the light-cured temporal denture base resin handle, which itself was bonded to the detection target. The subject’s head was then fixed in a head-retention bracket, and the splint was seated flush against the lower dentition. Lastly, the subject was asked to make open-close, protrusion, lateral, habitual chewing and swallowing movements while the system simultaneously collected target point spatial data (Fig 3).

#### Preliminary evaluation of system accuracy

**Fig 4:**
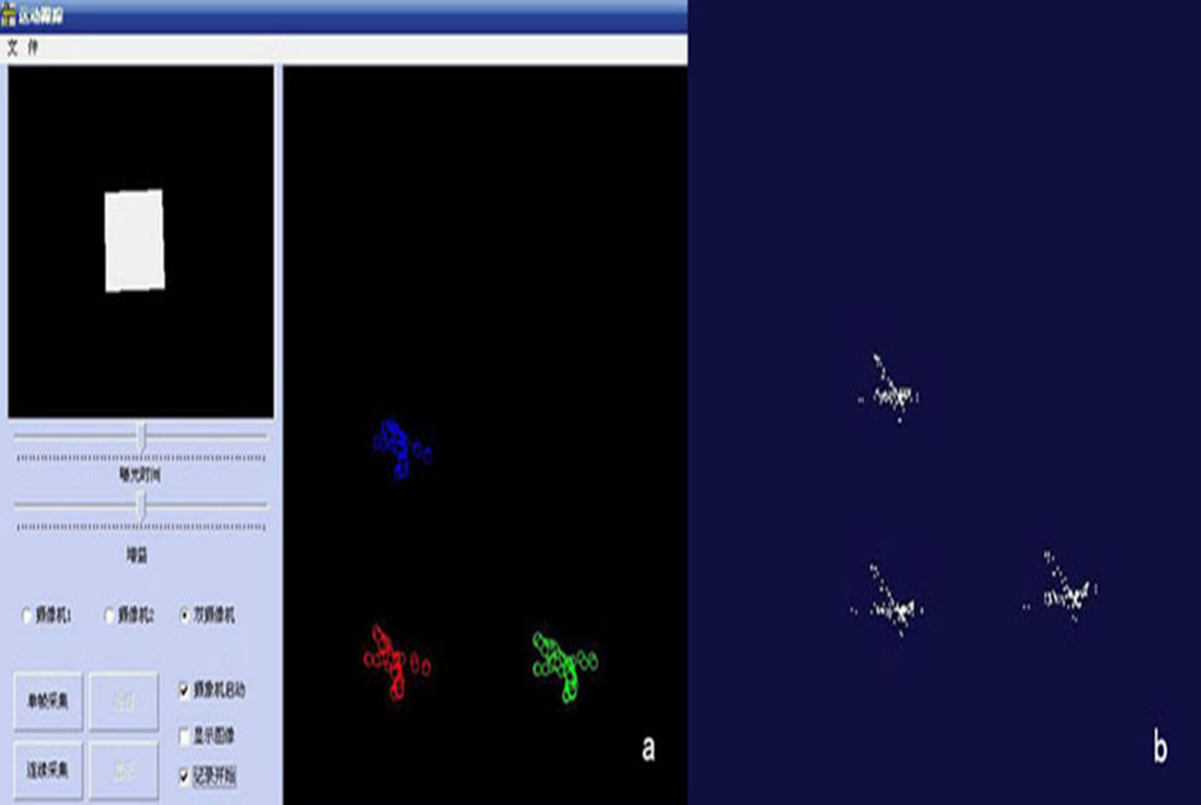
System detection precision analysis.

A platform for precise target detection was developed to validate the system algorithm's results and analyze the accuracy of mapping and divergence of the network. The detection target was designed with a smallest side length of 5 mm. When affixed flatly to a detection platform and the coordinates installed into the detection set in the software, the true coordinate value of Z was 0 and the true coordinate values of X and Y were multiples of 5 mm. The 30 cross point coordinate values of XYZ were then put into the SPSS statistical analysis software and analyzed using a single sample t-test. (Fig 4) To assess the accuracy of measurement based on actual subject movement, coordinate values of the 30 cross points on the detection target and the actual coordinate values were compared using a paired t-test.

## Results

### Three-dimensional reconstruction results of the trajectories of the lower dentition and the detection target

**Fig 5:**
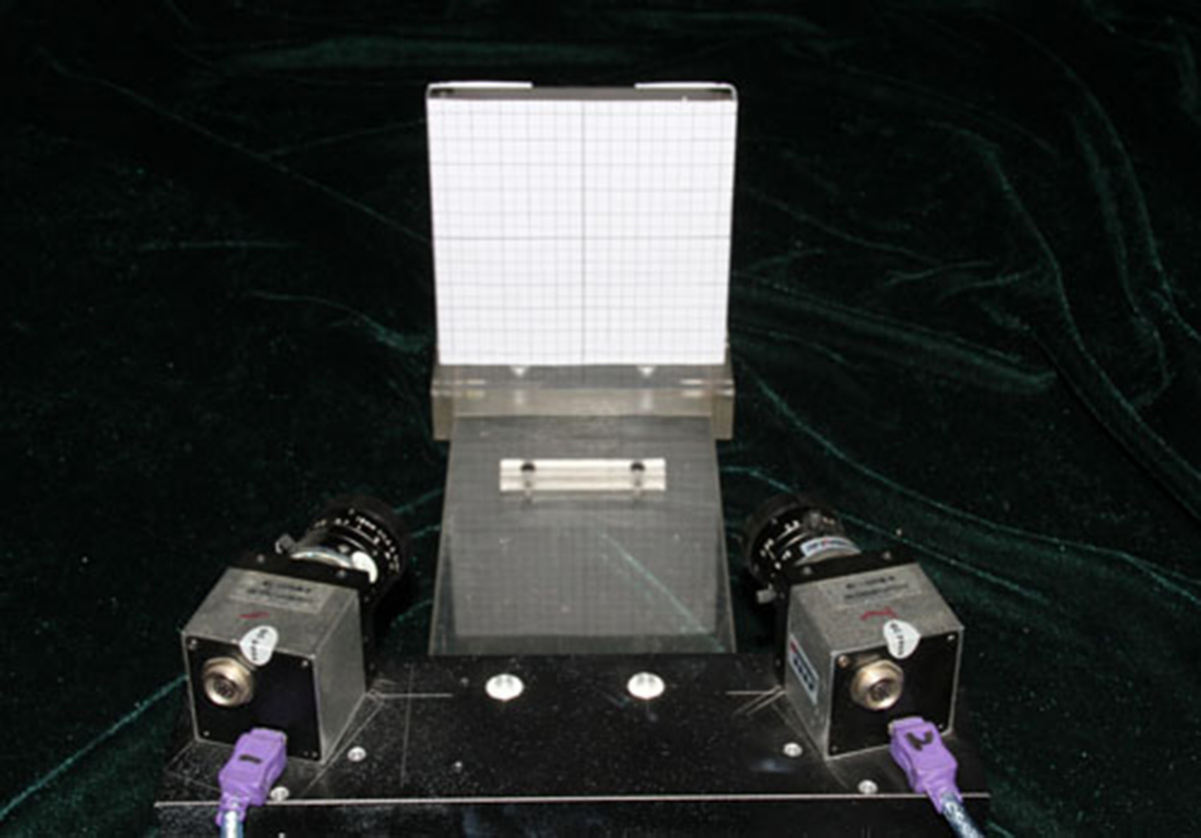
Consistency of subject movement and derived trajectory curve. (a) Subject’s actual movements; (b) Trajectory curve created by Imageware software.

The TXT file containing trajectory data was opened in the Imageware software, then the point cloud curve tool was used to create a curve of the three targets’ trajectories, in particular the three-dimensional trajectory curve of the whole mandible. In this experiment, the trajectories of open-close, protrusion, lateral, habitual chewing and swallowing movements were recorded. Upon visual assessment, each trajectory curve was consistent with the subject’s actual movements in both shape and direction (Fig 5).

### Accuracy of evaluation

Coordinate values of the 30 cross points on the detection target, the actual coordinate values and the measurement results are shown in S1 Table.

The values of mean ± SD and a paired t-test of P between the measurement coordinate values and actual values were as follows: X axis: -0.0037 ± 0.02953, P = 0.502; Y axis: 0.0037 ± 0.05242, P = 0.704; and Z axis: 0.0007 ± 0.06040, P = 0.952. The t-test result showed that the coordinate values of the 30 cross points were considered statistically no significant. (P<0.05).

## Discussion

Movement tracking refers to the continuous recording of the position and orientation of an object relative to a rigid body over a period of time. For the rigid body, its position and orientation information can be expressed by three-dimensional vector mapping. Two coordinate systems, the world coordinate system and the object coordinate system, should be established before tracking. The object moves within the world coordinate system, so the location information can be expressed by the object coordinate system’s origin coordinates within the world coordinate system. Furthermore, the orientation information can be expressed by the projection vectors of the three axes of the object coordinate system within the world coordinate system. Thus, the movement of the object can be expressed by four three-dimensional vectors. Put more simply, movement tracking is the continuous recording of these four three-dimensional vectors over a certain period of time.

Movement tracking technology has important applications in various fields, such as space technology, where it can be used to study the movement characteristics of space targets or in aerospace controlling; in the military, where it can be applied to develop precision strikes; and in biological research, where it can be used to study biological migration patterns. The purpose of this study was to utilize this technology for tracking mandibular movement.

Various specialized technologies can be used for movement tracking, such as inertial technology, radar technology, optical technology and ultrasonic technology. Military uses of inertial technology include the recording of information related to position and orientation of movement using gyroscopes and accelerometers installed on aircraft, submarines, satellites and missiles. While inertial technology is typically unaffected by external interference, it is also typically applied to the measurement of large object movement and is therefore not suitable for oral medical application. While ultrasonic technology can be used to study mandibular movement with high accuracy [12], other methods, such as X-ray measurement, may be too harmful or may otherwise be inappropriate for this application [13].

With recent improvements in computer and digital camera technology, increasing numbers of 3D measurements based on computer binocular vision are being collected. Considering the precision, speed and cost of this process, recording mandibular movement may now be more feasible than it has been in the past. Moreover, with the development of related technologies, wider general use may also be possible. In taking another step toward this goal, we have therefore developed a prototype system of three-dimensional recording of mandibular movement based on two-dimensional image feature extraction.

In this system, the detection target was fixed with the mandibular dentition to allow for the simultaneous recording of the position and orientation of the mandible and lower dentition. The data in the camera coordinate system were converted into their corresponding spatial coordinate system according to the neural network algorithm. The computer binocular vision device was composed of two digital cameras fixed on a plate, and the optical axis of the two camera lenses crossed at a single focal point. To ensure that the entire measurement field could be imaged clearly, the focal length of the lenses and the apertures were adjusted, and the position and orientation of the camera on the board was adjusted and secured. The cameras were then calibrated to establish a spacial Cartesian coordinate system (world coordinate system) in the measurement field while simultaneously assessing the relationship between a pair of image pixels (xp, yp) with two points (xs, ys, zs; xe, ye, ze) to determine a spatial line.

The combination of detection target, lower dentition and the mandible constituted the tracking target in this study. To record changes in the target's position and orientation, landmarks identified in the target surface should be established. These landmarks represent the object coordinate system on the target. In principle, with a flat target, three points that are not in a straight line should suffice.

However, considering the speed of the software, this scheme is still inefficient. To recognize and follow a single black point on a large white background, the computer must examine all pixels continuously, which results in a lower speed of calculation. We therefore designed a specialized black/white two-dimensional pattern to improve this recognition speed.

The three-dimensional trajectory of the lower dentition was obtained quickly and relatively accurately based on the special two-dimensional and binocular stereo data. Furthermore, the digital simulation of the occlusal movement based on the trajectory points was realized using a least squares error registration algorithm. Notably, the simulation accuracy met the clinical needs for designing fixed prostheses. The mandibular movement output data included the key parameter values, two-dimensional trajectory data and three-dimensional trajectory data, which were stored in TXT or STL format, respectively. The data were then inputted into the virtual articulation software. To make a pathological and physical diagnosis of temporomandibular joint movements, including condylar and incisal point, centroid, border, language, chewing, joint disc and chewing muscle movements, the individual characteristics of mandibular movement can be simulated digitally through the rectification of dentition data and mandibular three-dimensional data. Based on the data in this study, the developed measurement system can be used to design a clinically acceptable occlusal surface of a dental prosthesis with a measurement accuracy of ± 0.1 mm. In spite of this, it is necessary to make further research to confirm the clinical applications of the method.

## Supporting Information

S1 Table Coordinate values of the 30 cross points on the detection target, actual coordinate values and measurement results. (XLS)
